# Facilitating Positive Spillover Effects: New Insights From a Mixed-Methods Approach Exploring Factors Enabling People to Live More Sustainable Lifestyles

**DOI:** 10.3389/fpsyg.2018.02699

**Published:** 2019-01-31

**Authors:** Patrick Elf, Birgitta Gatersleben, Ian Christie

**Affiliations:** ^1^Centre for Environment and Sustainability, University of Surrey, Guildford, United Kingdom; ^2^School of Psychology, University of Surrey, Guildford, United Kingdom

**Keywords:** spillover, sustainable lifestyles, identity, longitudinal, pro-environmental behavior

## Abstract

Positive spillover occurs when changes in one behavior influence changes in subsequent behaviors. Evidence for such spillover and an understanding of when and how it may occur are still limited. This paper presents findings of a 1-year longitudinal behavior change project led by a commercial retailer in the United Kingdom and Ireland to examine behavior change and potential spillover of pro-environmental behavior, and how this may be associated with changes in environmental identity and perceptions of ease and affordability as well as perceptions of how participation in the project has helped support behavior change. We draw on both quantitative and qualitative data. Study 1 examines quantitative data from the experimental and a matched control group. Study 2 reports qualitative findings from a follow up interview study with participants of the experimental group. As expected, we found significant changes in reported pro-environmental behavior and identity in the experimental group as well as some indications of behavioral spillover. These changes were not significantly associated with changes in environmental identity. The interviews suggested that group dynamics played an important role in facilitating a sense of efficacy and promoting sustained behavior change and spillover. Moreover, the support by a trusted entity was deemed to be of crucial importance.

## Introduction

Tackling anthropogenic climate change and other major challenges of human impact on our ecological life support systems cannot be achieved without behavioral change by individuals and communities (Capstick et al., 2015). Over the last decades the social sciences have made significant advances in research targeting ‘environmentally friendly’ behaviors (e.g., Kollmuss and Agyeman, 2002). With mounting environmental pressures and climate change impacts already happening across the world (Intergovernmental Panel on Climate Change [IPCC], 2014), further approaches to sustainable consumption are needed to establish more sustainable lifestyles in which people act sustainable across a wide range of possible behavioral areas (Thøgersen, 1999).

However, research has shown that changing behaviors poses great difficulties (Whitmarsh, 2009). Moreover, changing entire lifestyles is more difficult than targeting single behaviors or behavioral categories. Modern lifestyles consist of a highly complex mesh of moral, practical and cultural *commitments* to certain practices of consumption, and often involve very limited *capabilities* for self-directed change (see e.g., Nussbaum, 2011). For example, whereas people often hold a positive attitude toward pro-environmental behaviors (PEBs) such as recycling, a lack of recycling infrastructure and a supportive cultural context can present difficult barriers to realizing such behavior in everyday life.

At the same time individuals experience an ever-growing range of consumption and informational choices increasingly influenced by stimuli triggered by companies and governments (Thaler and Sunstein, 2008) with a major impact on both opportunities and abilities people have to shape their lifestyles, and to pursue more sustainable lifestyles. As noted by Jackson (2008), trying to engage in sustainable lifestyles thus throws up significant challenges for most people, sometimes leaving them with a feeling of being ‘locked-in’ (Sanne, 2002).

One way to approach the challenge of promoting more sustainable lifestyles is by studying behavioral spillover (Capstick et al., 2015). Lifestyles usually consist of a wide range of behavioral patterns, interests, beliefs, values and identities, among others. Theoretically, allowing behavior to spill from one behavior over to another can, potentially, trigger a chain-reaction that eventually changes entire lifestyles.

By positive spillover we refer to the adoption of further PEB, over and above the behavior targeted in a given intervention, and ideally extending beyond the duration of the intervention project. This means that sustainable lifestyles, characterized by consistent behavioral patterns with a relatively low environmental impact, can be achieved through behavior changes in both specific targeted behaviors and contexts which subsequently influence other behaviors.

This paper evaluates findings from a longitudinal behavior change project to examine how we might be able to promote more sustainable lifestyles through behaviors change and positive spillover.

### Behavioral Spillover Effects

Behavioral spillover refers to the process where adoption of one behavior spills over into the adoption of another. Spillover effects are often seen to occur as a result of changes in motivation or preferences at the individual level that result from the adoption of a new behavior and impacts on further behavioral outcomes (Truelove et al., 2014). Spillovers can be both positive and negative (Truelove et al., 2014; Dolan and Galizzi, 2015). Whereas *positive* spillover describe the process of one behavior leading to a second behavior that is in line with the initial intervention, and thus follows a certain consistency (assimilation), *negative* spillovers describe the process of a subsequent behavior that is inconsistent with the previous one. Negative spillover may occur when the initial behavior was perceived as too easy or costless since it has been suggested to be less reflective of one’s motivations (Truelove et al., 2014). Another, perhaps more common negative spillover effect occurs when individuals compensate for the initial behavior (e.g., Bargh et al., 2001; Gneezy et al., 2011; Dolan and Galizzi, 2015). Here, one potential explanation for negative spillover effects frequently offered by the literature is that of *moral licensing* (For a recent meta-analysis see: Mazar and Zhong, 2010; Blanken et al., 2015). Moral licensing refers to a process where adoption of one moral behavior results into a decreased likelihood of adoption of another. The idea is that the adoption of one moral behavior reduces motivation to engage in another, or may even increase the likelihood someone may adopt deviant behavior, because people feel they have “done their bit.” Another form of negative spillover is the so-called *rebound* (Druckman et al., 2011), or *backfire effect* (Jenkins et al., 2011) where financial savings achieved through one type of PEB are subsequently spent on environmentally damaging behaviors which may sometimes cancel out (rebound), or even exceed (backfire) any environmental savings.

Over the last 20 years empirical research into spillover effects has made significant advances. It has been proposed that behavioral spillover theoretically has the potential to support people in their transition toward sustainable lifestyles (Whitmarsh and O’Neill, 2010; Capstick et al., 2015). However, the findings of this research are varied, and spillover is difficult to detect. In an early study using a correlational design, Thøgersen (1999) found little evidence for spontaneous spillover. He did find a small but significant effect of both positive and negative spillover, but without increasing the overall predictability of subsequent PEB. However, he did find that spillover was more likely when behaviors were perceived to be more similar. In a more recent study with a similar design, Lanzini and Thøgersen (2014) found positive spillover from ‘green’ purchasing to other PEB. Examining the role of different categories on positive spillover effects, Thøgersen and Ölander (2003) reported that spatial and temporally similar PEB seem to show stronger correlations than behaviors within different taxonomic categories. These findings were partly confirmed by a recent study by Margetts and Kashima (2016) in which the authors found that behaviors drawing on similar resources (e.g., time and/or money) had a stronger effect on the magnitude of spillover effects to occur. Existing evidence for positive spillover effects were mostly found for low-cost behaviors that are ‘simple and painless’ (Thøgersen and Crompton, 2009). However, in a recent study by Lauren et al. (2016), the authors note that easy behaviors can lead to a strengthened intention to enact more difficult behaviors in the future through an increased sense of self-efficacy. This is in line with what Deci (1975; Ryan and Deci, 2017, p. 152) calls “optimal challenge” where a first less onerous task demands a subsequent, more challenging task leading to new capabilities. In contrast, van der Werff et al. (2014) demonstrated that more difficult behaviors can function as stronger signals of an environmental identity and thereby promote positive spillover.

### Identity, and Its Influence on Pro-environmental Behaviors and Lifestyles

Identities play important roles in guiding behaviors in everyday life. Self-identities provide an answer to the both explicit and implicit question of “Who are you?” (Vignoles et al., 2011). According to MacAdams (1995) identities encompass physical attributes, values, goals, behavior and traits together with an individual’s personal narratives. The significance of identities on human behavior is highlighted in numerous theories such as Self-Completion Theory (Wicklund and Gollwitzer, 1985), Identity Theory (Stryker, 1968, 1980; Burke and Reitzes, 1991), and Identity Based Motivation Theory (Oyserman and Lewis, 2017). Besides their conceptual differences, what these theories have in common is the assumption that humans have an inherent tendency to seek consistency in outlook and action.

According to Oyserman and Lewis (2017), identities “[a]re central to understanding motivation because people prefer to act and make meaning through the lens of their identities.” They are thus crucial for the transition to more sustainable lifestyles. Moreover, identities carry action- and procedural-readiness, and are cued by situations and the availability of awareness (Oyserman, 2009). Identity is a highly relevant concept for studying spillover, as the notion that people strive for consistency (Festinger, 1957) also serves as basis for the work on spillover effects (Thøgersen, 2004).

For the purpose of this paper we follow Oyserman et al. (2012) definition of identities as “traits and characteristics, social relations, roles, and social group memberships that define who one is.” This definition, as many others, highlight the important of social relationships, social norms and roles for identities. Identities reflect how people see themselves in relation to other people.

In summary, as suggested by Gatersleben et al. (2014), identities can be understood as stable factors that have the potential to transcend spatial and temporal situations and support behavioral consistency and potential spillover. As suggested by the literature, identity can strengthen perceived efficacy and the sense of belonging, and shift identity standards (Burke, 2006) toward a more pro-environmental understanding of oneself.

### The Role of Identity as a Potential Driver for Positive Spillover Effects

Whitmarsh and O’Neill (2010) highlight the crucial role of identity, providing compelling evidence that self-identity operates as a significant behavioral determinant beyond usual variables for carbon offsetting behaviors. Additionally, van der Werff et al. (2014) showed in a series of studies that simply reminding people of their past PEB led, on average, to a strengthened pro-environmental identity and, in turn, to an increased probability of engaging in further PEB. Lacasse (2016) also found that people performed behaviors according to their past-behaviors when they were reminded of them. In addition, the study showed that labeling people with a pro-environmental identity had a stronger positive spillover effect than inducing guilt. On the other hand, Poortinga et al. (2013), found no spillover from increased use of reusable shopping bags (in response to a charge for disposable bags) and other PEBs. However, the research did find an increase in self-reported pro-environmental *identity*. It can be speculated that the absence of a positive spillover effect can be explained by the fact that the behavior was externally regulated (the bag charge), leading to a sense of compliance through the introduction of the new law rather than autonomously enacted behavior (Ryan and Deci, 2017, pp. 191, 226).

### Current Research

The aim of our research is two-fold. Firstly, we examine changes in reported PEB, environmental identities and perceptions of ease and affordability among participants of a longitudinal behavior change project. Second, we examined the consistency of behavior and explored potential spillover of behaviors and explored how participation in the project may have supported (or not) such spillover. We draw on both quantitative and qualitative data from the so-called Live Lagom behavior change project executed by a commercial retailer in the United Kingdom and Ireland.

The word *lagom* is sometimes said to describe the Swedish way of life. Loosely translated it means ‘just the right amount’ or ‘balance.’ It is an alternative approach to sustainable lifestyles that emphasises the idea of sufficiency.

The project involved a continuous interaction between the participant (i.e., customer) and retailer (i.e., lifestyle change support system), with the aim to allow customers to overcome barriers to more sustainable lifestyles at home and create a movement of like-minded people. Based on the notion that simple education is no longer the dominant approach, it applied a wide range of behavior change techniques (see Supplementary Data Sheet 1, Appendix A) grounded in existing literature (e.g., Abraham and Michie, 2008).

The initial induction workshop, together with information material in the form of a brochure intended to generate an improved understanding and awareness of sustainability related issues such as resource (over-) consumption, among others. After the participants received their products, they engaged in a number of interventions such as workshops, online awareness-raising activities, and reflective blog writings (for an overview please refer to Supplementary Data Sheet 1, Appendix A). Here, the applied interventions targeted a wide range of behaviors. The bi-monthly workshops organized by the retailer targeted first and foremost behaviors that the retailer was able to support participants with through their product range (e.g., energy savings through an LED range, food storage containers). At the same time, informing participants about product labeling can be considered to be transferable so that some of the inventions potentially triggered behavior change that went beyond the retailer’s own area of expertise.

Between the workshops the closed Facebook group allowed participants across different locations to communicate. This, together with an online question and answer session on energy savings with an industry expert intended to allow participants to engage in further PEB changes. Participants were then able to reflect on their process in their blog posts they wrote at different stages during the project.

## Study 1: Quantitative Study

The quantitative survey study examined whether participation in the project resulted in changes in PEB, identity and perceptions, and how these were related. We hypothesized that reported PEB would increase more in the experimental group than in the control group. To gain insight into potential spillover we examined how different behavior changes were related (those targeted and those not targeted by the project intervention). To gain insight into potential rebound we examined how people said they had spent money they had saved by adopting sustainable behaviors. We further examined whether behavior changes were associated with changes in reported environmental identity and perceptions of desirability and ease and affordability.

### Design

Quantitative data were collected from a participant and a control group through a baseline questionnaire in November 2016 and through a subsequent follow-up questionnaire during July 2017. The project had an extended focus, and more detail can be found in Supplementary Data Sheet 1, Appendix A.

### Sample and Procedure

The participant sample was recruited by the retailer through the company’s loyalty program on the basis of location (to ensure participants could attend relevant workshops and other interventions – See Supplementary Data Sheet 1, Appendix A) and perceived interest in making changes to their current lifestyles. Each participating household received a voucher to the value of £300: they were allowed to spend this on a range of products that were categorized as sustainable (i.e., the products have the potential to support participants to engage in sustainable lifestyles). In all, 100 participants were recruited in 19 different locations across the United Kingdom and Ireland according to store locations of the retailer. A control group was then recruited by a market research company who matched the control sample to the participant sample. In total 1,000 people in the control group completed the baseline survey but only 170 respondents completed both baseline and follow up survey and were included in the analyses reported here. After cleaning the data and removing missing or non-matching data, a sample including 152 responses in the control group and 99 in the experimental group remained. In both groups there were more females (67% in the experimental group and 72% in the control group). In the control group 30% of the respondents were 35 or younger, 43% between 35 and 44, and 28% 45 or older.

### Measures

All respondents completed a large survey including a wide range of questions on PEBs, environmental attitudes, values and identities. The analyses in this paper focus on the following parts of the survey only.

#### Desirable, Easy, and Affordable

Respondents were asked to rate on a scale from 1 (not at all) to 5 (very much so) to what extent they believe it was desirable, easy and affordable to live a sustainable lifestyle.

#### Identity

To measure identity respondents were asked four questions. How important (1 = extremely important, 5 = not at all important) is it to your sense of self: to try to live a sustainable lifestyle; […] that other people think of you as someone who lives sustainably; […] that those people living with you practice sustainable behaviors; […]. The items were combined into one identity variable by calculating the mean score across the three items for time 1 (α = 0.79) and time 2 (α = 0.82). At the end of the project participants were also asked to what extent they felt like a Lagomer (1 = not at all, 100 = completely).

#### Pro-environmental Behavior

Respondents were asked how often (point 1 = never, 5 = always) they enacted ten PEBs: switch off lights in rooms that aren’t being used, switch off appliances and not leave them on standby, maintain, repair and/or “upcycle” things, avoid food waste, for example by planning meals ahead, measuring the right portions, using containers to prolong the life of food, or cooking with leftovers; use product labeling to help you choose the most energy- and water-efficient products; choose fairly traded, eco-labeled and independently certified foods, clothing, etc.; buy second hand or recycled products; hire, share and lend products instead of buying them; use reusable shopping bags; walk or take the bike instead of the car for short journeys. A new variable was created combining ten (never-always) of these behavior variables into one scale (α = 0.75 for T1 and 0.76 for T2).

#### Rebound

To gain insight into potential rebound effects, respondents were asked whether they thought they had saved money during the project by saving energy and water. Here, 25% of the respondents stated they felt they had saved ‘a lot’ of money on electricity savings, and 42% said they had saved ‘a little.’ 14% said they had saved ‘a lot’ on gas bills, and 38% said they had saved ‘a little.’ In terms of water savings, 8% stated they had saved ‘a lot’ on water bills, and 32% said they had saved ‘a little.’ Finally, 27% said they had saved ‘a lot’ on food bills whereas 39% thought they had saved ‘a little.’

### Results

#### Desirable, Easy, and Affordable

**FIGURE 1:**
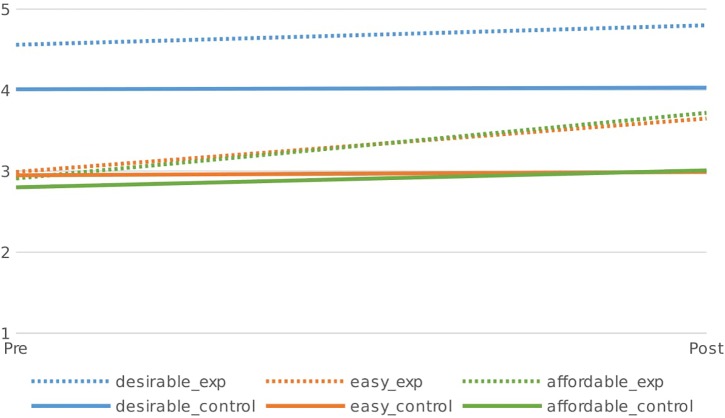
Perceived desirability, ease and affordability of sustainable living at the start of the Project and 8 months later by respondents in the control and experimental condition (1 = low, 5 = high).

Participating in the project had a significant positive effect on respondent’s perceptions. Perceptions of the desirability of sustainable living did not change significantly more in the experimental than in the control group [Wilks = 0.99, *F*(1,231) = 3.54, *p* = 0.06, η = 0.015]. This is perhaps due to a ceiling effect as perceptions were already very high. However, participants in the experimental group were significantly more likely than participants in the control group to see sustainable living as easier [Wilks = 0.94, *F*(1,231) = 15.91, *p* < 0.001, η = 0.064] and affordable [Wilks = 0.96, *F*(1,231) = 10.85, *p* = 0.001, η = 0.045] at time 2 than at time 1 (see Figure 1).

#### Identity

**FIGURE 2:**
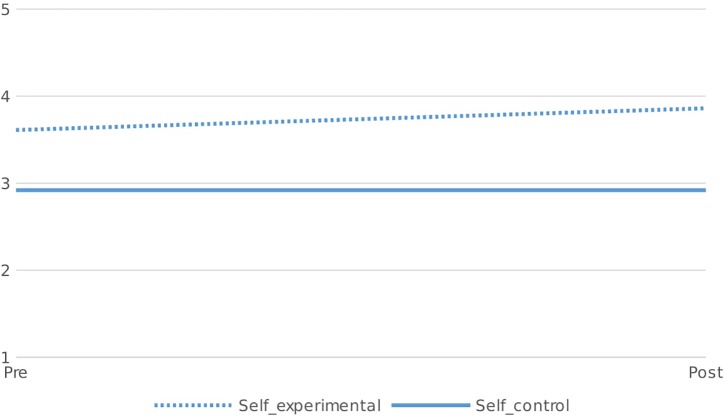
Environmental identity at the start of the Project and 8 months later by respondents in the control and experimental condition (1 = low, 5 = high).

At the start of the project participants were already more likely to see living sustainably as an important part of their identity. Yet, this difference increased further during the project (Figure 2). A significant interaction effect revealed that participants in the experimental group were significantly more likely than respondents in the control group to perceive living sustainably as important to their sense of self at the end of the project compared to the start of the project [interaction effect Wilks 0.98, *F*(1,231) = 4.65, *p* = 0.032, η = 0.020]. At the end of the project participants also tended to indicated that they felt like a Lagomer (*M* = 79, *SD* = 17) indicating that they had incorporated the project identity.

#### Behavior Change

Table 1 (left part) shows that all behaviors were adopted more at the end than at the start of the project. However, the changes were largest for “avoiding food waste” and “using labeling to buy more energy efficient products” and “eco-friendly products” (columns 2–4 of Table 1), behaviors targeted by the project interventions. However, behaviors that were not targeted also changed. The last three columns of Table 1 show that, as expected, change scores for each behavior (post minus baseline) were larger in the experimental than in the control group. In fact, for most behaviors there was no evidence for any behavior change in the control group (mean change scores were close to zero). The largest differences between the control and the experimental groups were found for the item on *avoiding food waste*, *using reusable shopping bags*, and *using labeling*.

**Table 1:** Changes in reported behaviors pre–post the intervention period, and differences between the experimental and control group in reported behavior changes.

	Changes in reported behavior			Differences in behavior change		
		
	Pre	Post		Experimental	Control	
				(*N* = 80)	(*N* = 152)	
	M	M		M	M	
	SD	SD	*t*	SD	SD	*t*
Lights	4.23	4.46	19.62***	0.51	0.07	7.51**
	0.93	0.84		0.83	1.02	
Standby	3.19	3.45	19.65***	0.61	0.06	11.12**
	1.27	1.23		1.06	1.13	
Maintain, repair upcycle	2.85	3.05	11.63**	0.49	0.04	6.57*
	1.21	1.13		1.04	1.16	
Avoid food waste	3.70	4.05	51.42***	1.11	-0.05	43.18***
	1.17	1.01		1.09	1.07	
Labeling energy	2.73	3.16	36.72***	1.05	0.11	16.89***
	1.35	1.35		1.14	1.50	
Labeling food clothes	3.70	4.05	51.42***	0.78	0.13	18.67***
	1.17	1.01		0.97	0.97	
Buy second hand	2.34	2.44	9.10**	0.44	-0.07	12.44**
	0.92	0.93		0.85	0.91	
Hire, share and borrow	1.90	2.07	13.18***	0.78	0.01	5.79*
	0.81	0.82		0.91	1.00	
Reusable shopping bags	4.26	4.48	10.23***	0.45	-0.11	25.97***
	0.88	0.89		0.76	0.79	
Walk or bike instead of car	3.11	3.27	8.30**	0.40	0.03	5.71*
	1.30	1.35		0.98	1.14	


*^∗^p < 0.05; ^∗∗^p < 0.01 Significance levels.*

#### Spillover

Table 1 showed that reported behavior changes spanned a wide area. Reported behavior changes of participants in the experimental group were strongest for behaviors that were more strongly linked to the project (reducing food waste, purchasing labeled products) but also for behaviors that were not addressed in the intervention (walking or cycling instead of using a car). These findings suggest spillover may have taken place. To explore this further correlations were computed between all behavior change scores. Table 2 shows these correlations for the experimental group (top) and the control group (bottom). The findings confirm that there are more significant correlations in the experimental group than in the control group, suggesting that behavior changes for one behavior were more likely to be associated with behavior change for another behavior in the experimental group. Correlations in the experimental group are also stronger, pointing to the same conclusion. Finally, in the experimental group changes in behaviors that were addressed in the intervention (reducing food waste, using labeling, sharing and repairing) as well as those that were not (walking and cycling) were correlated with a number of other behavior changes.

**Table 2:** Correlations between different behavior changes in the experimental and the control group.

	Light	Appl	Repair	Food waste	Label	Fair	Second hand	Share	Bags
**Experimental group**									
Lights	1								
Appliance	0.22	1							
Repair	0.35**	0.32**	1						
Food waste	0.17	0.12	0.36**	1					
Label	-0.04	0.21	0.16	0.21	1				
Fair	0.23*	0.20	0.16	0.22	0.20	1			
2^nd^ hand	0.02	0.02	0.34**	0.27*	0.10	0.06	1		
Share	0.16	0.06	0.45**	0.40**	0.23*	0.27*	0.43**	1	
Bags	0.31**	0.08	0.26*	0.17	0.19	0.16	0.34**	0.20	1
Walk bike	0.17	0.30**	0.28*	0.06	0.29**	0.23*	0.05	0.22*	0.11
**Control group**								
Lights	1								
Appliance	0.09	1							
Repair	0.02	0.16*	1						
Food waste	0.14	-0.03	0.08	1					
Label	0.02	0.14	0.33**	0.24**	1				
Fair	0.07	0.07	0.11	0.21**	0.39**	1			
2^nd^ hand	-0.11	0.14	0.14	0.15	0.18*	0.17*	1		
Share	0.03	-0.01	0.11	0.23**	0.16*	0.08	0.30**	1	
Bags	0.06	0.07	0.03	0.16	0.06	-0.03	0.02	0.00	1
Walk bike	0.07	-0.02	0.02	0.02	-0.01	-0.04	0.00	-0.08	0.10


*^∗^p < 0.05; ^∗∗^p < 0.01 Significance levels.*

#### Rebound

When asked what participants had done with the money savings resulting from the project, 22% of the respondents reported they had spent it on social events and trips (holidays (10%), visiting friends, weddings), 18% said it went toward savings (6%) or payment of household bills (12%). Interestingly, only 9% of the participants stated that they spent it on products to help them further cut down environmental impact. This was almost always on food containers, light bulbs or plants and seeds. Finally, 5% said they spent it on home improvements such as extensions, curtains, rugs and well as generic home improvements, in some cases to help energy saving.

#### Identity and Behavior Change

To examine whether changes in identity were associated with changes in PEB and rebound, we first created a new identity change score by calculating the difference between baseline and follow-up scores. The same was done for changes in perceived ease and affordability. Resulting scores could be negative (a reduction), zero (no change) or positive (an increase). Overall, sustainable identity became less salient for 34% of the respondents, stayed the same for 19% and became more salient for 47% of the respondents. 25% of the respondents thought it was less easy compared to 41% of the participants who thought it was easier to live sustainably after the project than before, for 34% it stayed the same. 45% thought it was more affordable, and 20% thought it was less affordable at the end than at the start of the project, for 35% it stayed the same.

**Table 3:** Correlations between changes in identity, perceptions of desirability, ease and affordability of sustainable behaviors, and changes in reported behaviors.

	Changes in			
	
	Identity	Desirable	Easy	Affordable
PEB	0.13^∗^	0.21**	0.27**	0.21**
Lights	0.12	0.13	0.17**	0.13*
Appliance	0.06	0.03	0.14*	0.16*
Repair	0.08	0.09	0.09	0.07
Food waste	0.03	0.13	0.15*	0.12
Label	0.07	0.17*	0.06	-0.00
Fair	0.15^∗^	0.16*	0.25**	0.18**
Second hand	0.10	0.14*	0.08	0.05
Share	-0.01	0.13	0.18**	0.18**
Bags	0.02	0.17*	0.13	0.13
Walk bike	0.08	-0.02	0.17**	0.11


*^∗^p < 0.05; ^∗∗^p < 0.01 Significance levels.*

Regression analyses were conducted to examine whether changes in reported PEB were associated with changes in reported identity and changes in the perception of how easy or difficult it is to adopt such behavior. For changes in reported PEB, a significant relationship was found. However, this effect was small, and only 6% of the variance in behavior change could be explained by changes in identity and perceptions [Adj *R*^2^ = 0.06; *F*(3,228) = 5.96, *p* = 0.001]. Moreover, only changes in perceived ease of the behavior was a significant predictor (β = 0.19, *p* = 0.023), whereas changes in identity (β = 0.10, *p* = 0.129) and perceived affordability (β = 0.06, *p* = 0.465) were not. Table 3 shows correlations for each of the behaviors separately. The table illustrates that increased perceptions of the desirability of PEB were associated with changes in consumer behaviors such as use of labeling, fair trade products and usage of reusable bags. This, however, was not related to energy saving behaviors such as turning lights and/or appliances off. Changes in perceived ease and affordability were related to a wider range of behaviors but least with low cost (or cost saving) behaviors such repairing things, using energy labeling, buying second hand and using reusable bags.

Although changes in identity did not related to changes in reported behavior, reported identity and perceptions at baseline were significant predictors of reported behavior at time 2 [Adj *R*^2^ = 0.22; *F*(3,228, 23.17), *p* < 0.001], with only pro-environmental identity being a significant predictor (β = 0.44, *p* < 0.001). Moreover, pro-environmental identity at time 1 was a significant predictor of behavior change [Adj *R*^2^ = 0.06, *F*(3,228) = 5.76, *p* = 0.001; β identity = 0.28, *p* < 0.001; β easy = -0.11, *p* = 0.213; β affordable = 0.02, *p* = 0.817].

Examining respondents in the experimental group only, a positive correlation was found between reported PEB at the end of the project and the extent to which respondents indicated they felt like a Lagomer (*r* = 0.36, *p* = 0.001). They also indicated that a Lagom lifestyle was a sustainable lifestyle [*M* = 87 (1–100), *SD* = 14]. Not surprisingly then, the Lagom identity was correlated with the environmental identity (*r* = 0.51, *p* < 0.001). However, this relationship was only significant for four out of the ten behaviors: switching off lights (*r* = 0.29, *p* < 0.001), repairing/upcycling (*r* = 0.41, *p* < 0.001), reducing food waste (*r* = 0.29, *p* < 0.01) and using energy labels (*r* = 0.27, *p* < 0.05) suggesting that the Lagom identity, maybe unsurprisingly, was “lived” first and foremost at home, and did not necessarily translate into other behaviors.

#### Rebound and Identity

In the experimental group, environmental identity became less salient for the 21% of the respondents, it stayed the same for 14% of the respondents and increased for 49%. To examine whether reported rebound was associated with changes in identity, χ^2^ tests were conducted. Unfortunately the sample size was too small to conduct reliable analyses. As we only had data from the experimental group and not all participants had answered the rebound question the samples were too small to conduct valid analyses (*n* = 54 in total, and too many cells, 66%, had expected count less than 0.5).

### Summary

As expected, respondents in the experimental group were significantly more likely than respondents in the control group to report an increase in behavior change and pro-environmental identities. Moreover, changes in behavior were more likely to be correlated in the experimental than in the control group, suggesting that there was some consistency of behavior change and potential spillover. Unfortunately it was not possible specifically to test spillover as we could not determine which behavior change took place first. A further limitation of the quantitative approach is that we can only study spillover for behaviors that were included in the survey. A follow up study is therefore needed to examine further behavior change.

Although reported perceptions, identities and behaviors all changed, the extent to which these changed were only marginally related to each other. Environmental identity predicted behavior change but changes in identity did not relate to changes in behavior. In summary the project was clearly successful in changing perceptions and reported behaviors but it is not entirely clear what may have contributed to these changes. A follow-up interview study was conducted to gain more insight into the processes of change and what may have contributed to successful behavior change and potential spillover.

## Study 2: Qualitative Interviews

The aim of the interview study was to shed further light on underlying factors that *enabled* participants to change a range of behaviors during their participation in the Live Lagom project. Hence, we examine *whether* spillover took place, and what motivated participants to engage with more PEBs.

### Methods

#### Participant Sample

Qualitative data were collected 9 months after the official end of the project during March 2018 by means of interviewing a sub-set of project participants. Potential interviewees (*n* = 44) were contacted on the basis of proximity to the first author’s locality due to practical and financial reasons. Seven householders agreed to participate in a semi-structured interview (Bryman, 2008, p. 439) in their home. All participants were ‘White British’ or ‘White other,’ female, and all except one had children. In two interviews male partners actively participated. The mean age was 41.1 years (ranging from 30 to 50) with a mean annual gross household income of around £40,000.

#### Procedure

The semi-structured interviews took place in four different locations across England and lasted between 45 and 90 min. Questions focused on changes in behaviors and factors enabling them whereas a high degree of flexibility was maintained to address potentially important findings. All interviews were transcribed verbatim and analyzed using a thematic analysis approach (Braun and Clarke, 2006; Bryman, 2008, pp. 554–555.). Thematic analysis allows the researchers to explore recurring topics between the participants and add explanatory power to the quantitative findings. No further incentive was provided for their time and participation.

The qualitative analysis was an iterative process, and included coding and categorization. Following Braun and Clarke’s (2006) six phases of thematic analyses, the first phase focused on becoming more familiar with the data. In a second step initial codes were generated. These were informed by a previous (similar) interview study conducted a year earlier with a different sample. First findings from this research, suggesting that identity *can* play a role in extended behavior change leading to spillover effects, were added where it seemed appropriate. In a third phase, the collated codes were used to build first themes that were subsequently reviewed in a fourth step before defining and naming them during the fifth phase. A sixth and final phase is the production of a report which builds the qualitative analysis of the research at hand.

**Table 4:** Respondents’ environmental identity, reported pro-environmental behavior, perceptions of desirability, ease and affordability of sustainable behavior, reported rebound and perceived achievements and barriers for further change.

	ID	PEB	Desirable	Easy	Affordable	Rebound	Biggest achievement	Most sust. behavior	Main barrier
BR 2-4	+1	+0.9	0	+2	+3	(NA)	New focus, now writing book	Live without plastic No new clothes	Cost
BR 2-6	+1	0	0	+2	+2	Savings	Growing own food	Not sure	Availability
NOT2-1	+0.4	+0.2	0	+1	0	Holiday	Family more mindful	Nappies	Time
NOR2-1	+0.7	+0.6	0	+1	+2	Bills	Made home more efficient	Nappies	Culture Cost
RE2-1	+1	+0.7	+2	+1	+1	Holiday	Energy waste	Energy waste	Time
RE2-2	0	+0.6	0	0	0	(NA)	Organized/tidy	Greener car	Cost
RE2-3	0	+0.6	0	+2	+2	Savings	Saved energy reduced cost	No second car	Cost


### Results

Table 4 shows the respondents’ answers to the key questions discussed in the quantitative section. It also shows how the participants responded to some further exploratory questions that aimed to gain further insight into their behavior changes and perceptions. The table illustrates the interviewees’ varied responses to the intervention. Behavior change was stronger for some than for other participants, as were changes in identity. Respondents RE2.2 and RE2.3 changed the least.

#### Results: Thematic Analysis

In addition, the thematic analysis uncovered a number of themes that provided insight into the ways in which participating in the project supported sustainable living. The first theme described below discusses evidence for behavior change and spillover and combines data from the qualitative and quantitative parts of the study. The following themes focus on perceptions of the ways in which project participation has supported behavior change: behavior change and spillover, support, belonging, identity, and structural barriers to making changes.

#### Behavior Change and Spillover

The quantitative findings had already provided evidence for reported behavior changes, yet, as in most spillover studies, demonstrating a strong spillover effect was more problematic. In line with the quantitative findings, interviewees were more likely to report a strong engagement with a range of PEBs at the end of the project compared to the start. Looking at the reported behavior changes on the quantitative survey for each of the interview respondents (Supplementary Data Sheet 1, Appendix B) suggests that behaviors enacted at home changed more than behaviors outside the households. These findings indicate two things. Firstly, that behaviors that were targeted by interventions (Supplementary Data Sheet 1, Appendix A) were mostly successful, and, secondly, that behaviors were more successfully changed when interviewees felt more in control of them. Moving from top to bottom, the table illustrates the difficulty to secure behavioral consistency across domains with the high PEB mean scores (i.e., the green areas) occurring much more on the top (i.e., in household behaviors).

**Table 5:** Overview of self-reported spillover-effects and barriers to further positive spillovers.

Participant	Behavior 1	Behavior 2 (i.e., positive spillover)	Barrier to further positive spillovers
Br-4	• Saving energy,	• Zero waste	• Financial means
	• Avoid food waste	• Plastic free	
		• Grow vegetables	
Br-6	• Saving energy	• Plastic avoidance	• Travel for job
		• Grow own	• Living situation
Nor-2	• Saving energy	• Improve recycling further	• Structural factors
		• Dry clothes on clothes airer	
Not-1	• Growing food	• Waste avoidance (e.g.: special bee wax sandwich wrap)	• Financial means
	• Saving energy		
Re-1	• Avoid food waste	• Waste avoidance (e.g., plastics)	• Structural factors
	• Save energy	• Using rechargeable batteries	• Lack of support from government (renewable energy)
		• Do own washing products	
Re-2	• Decluttering	• Energy savings	• Lack of interest and motivation
Re-3	• Save energy	• Being more mindful: reusing more when traveling	• About to move house soon
	• Avoid food waste		• Structural factor (e.g., recycling facilities)


The interviews also found evidence for self-reported spillover. Table 5 provides an overview of participants’ initially targeted behaviors, any reported positive spillover and reported reasons why no spillover had taken place. The table suggests that positive spillover occurred in the interview group. For example, a female participant from the southeast of England reported that the family initially intended to save energy and reduce their food waste. This, subsequently, led her to reuse her towels more when she traveled for work and try to use reusable water bottles instead of buying new ones, following an increase in awareness.

What follows is an overview of findings from the interviews conducted 9 months after the end of the project explaining in more detail the different roles of factors that influenced behavior change and positive spillover factors.

#### The Role of Support and Motivation for Behavioral Changes

Participants mainly described their motivation for applying to the project in terms of *support*, indicating both a willingness and an openness to change their existing lifestyles. Through entering into the project they hoped to receive help that would allow them to overcome barriers such as a lack of continuous motivation and awareness:

“It [the reason for applying] was- if there was any way to improve it and to make it more eco-efficient and to, you know, minimize impact we were having, that was really-…that’s quite important to us.” (Nor-2)

“It [taking part in the project] would give us a little bit of a push, if that makes sense, to kind of like…rethink of how we were living our lives here and we kind of needed that push to get us to be able to like review and…and, umm…think about how we can be more sustainable.” (Re-1)

As presented in Table 4, all except one participants reported no change for ‘desirability.’ The ceiling effect, already described in the quantitative analysis, provides a potential explanation here. At the same time, it suggest that the participant group had a naturally strong interest in changing their lifestyles, equipping them with an initial motivation that perhaps served as a fertile ground for further behavior changes, and/or, in other words, potential spillover effects.

Indeed, analysis on the behavioral changes also provide additional insights into the role of the retailer as *Lifestyle Change Support System* in the process. The facilitation of both the interventions and an environment that allows participants to engage in more PEB was considered to be of great importance to motivate participants to engage in further PEB as part of sustainable lifestyles:

“And I think just having someone to say ‘look, set it up like this. It will be easy to do everything.’ And then maybe a knock-on effect, isn’t it? To go through and say ‘oh, okay, that’s easy. Now let’s see if I can tackle this, or this, or this.”’ (Re-2)

Furthermore, the new relationship between the participants and the program, which we characterize as joint engagement in a *Lifestyle Change Support System*, resulted in a sense of *commitment* to enact newly developed capabilities and, eventually, change their lifestyles to more sustainable alternatives. The retailer refrained to inflict a sense of guilt to enact more PEB, nor did they directly remind participants of an earlier expressed pro-environmental identity. The resulting relationship between retailer and participating households had strong implications in participants’ motivation to change their lifestyles:

“You know, it’s not like an actively, or a contractual relationship or I signed something like ‘you must do this’ but I think it is the conscious realization that you are participating in a project and that you actively want to make these changes and that you are getting the support. (…) Yeah, it sort of is like ‘well, yeah I need to do this because they have done that.’ Because they care and because they want people to change. So yeah, we want to be those people that do change.” (Re-2)

This finding points toward a successful facilitation of what Ryan and Deci (2017, pp. 99, 617) call *need-supportive contexts* in which people have the opportunity to execute existing, and stretch newly developed, capabilities. Moreover, they suggest that within these environments people are much more likely to experience a process of internalization in which values, beliefs, or behavioral regulations from external sources, such as other participants and the Lifestyle Change Support System are taken in, and, eventually, transformed into the participant’s own.

#### The Role of Belonging to a Like-Minded Group for Positive Spillover Effects

Another main supporting factor facilitated by the retailer was the creation of a group of like-minded people that eventually bonded. The involvement in the group provided supporting mechanism that especially affected two important outcomes. Indeed, group membership plays a significant role. Identifying with a certain group, can have far-reaching effects on one’s belief systems, actions and motivations.

In the case of Live Lagom, it engaged participants to explore further PEB they did not initially intend to change, and, secondly, a strengthened sense of relatedness. For example, when prompted if they only focused on a certain goal, participants expanded on the process of how behaviors spilled over:

“Yeah, it expanded beyond that. People involved in the programme were able to help us to, well-…like, you can also do this and this and this. And we were like, ‘yeah, that’s a great idea, we can do that.”’ (Not-1)

Through the interaction with other participants belonging to the Live Lagom group, participants thus engaged in tasks that were readily but not easily ovecome, and thus offered ‘optimal challenges’ (Ryan and Deci, 2017, p. 448) which resulted in an increased sense of efficacy and nurtured an intrinsic motivation to engage in further behavior changes.

Overall, a general sense of belonging was seen of great significance, motivating participants to explore further behaviors that were initially not targeted – in other words, spillover activities:

“You gonna have these friends and they gonna think the same things and it’s gonna be ‘yes come on, let’s save it all.’ And we’ve been online going ‘does this-…taking pictures of packaging and can you recycle this, and can this go in the back?’ And trying to work out if you can or not (laughs). It is a minefield of plastic packaging out there. The film-type stuff. I have no idea (laughs). We just trying our best.” (Nor-2).

#### The Role of a Salient Pro-environmental Identity

As highlighted earlier, one main problem of engaging in sustainable lifestyles is a lack of consistency, which is also apparent in spillover studies. Here, establishing identity has been proposed to offer a potential way to generate commitments that can overcome this inconsistency. For the paper at hand, the increase in pro-environmental identity examined by the quantitative analysis was also apparent in the qualitative analysis in the form of sustainable behavioral outputs. For example, when asked if they would identify as sustainable citizens or, alternatively, with the Lagom project, most participants shied away from applying an identity label to themselves:

“There is no point to like self-describing myself. But I would say that it has made a distinct in our attitude about things…and we are very, very, you know-…just because I don’t describe myself as a Lagomer doesn’t mean that it didn’t have a massive impact on me or (name husband) or on our family.” (Re-2)

Although participants did not feel comfortable labeling themselves explicitly the qualitative analysis suggested that the idea of living a *lagom* lifestyle was integrated in the sense of self of the participants. For example, participating households anchored the *lagom* concept as a framework for sustainable living:

“I think it [lagom] became a word for our kids in the house as well. The kids would make a comment like ‘oh, I am being lagom.’ Or ‘I *lagomed’* my lunch. Like it was a verb. (…) I mean, I think carrying a catch-phrase helps you to keep it in your mind and is playful and sort of like *I am on that team.*” (Not-2)

What happened here can be described as a combination of two socio-psychological processes forming a social representation (Moscovici, 1984, 1988). Whereas the first, anchoring, reflects categorizing unfamiliar objects through comparing them with already existing, familiar and culturally accessible objects, the second, objectification, transforms these unfamiliar concepts into concrete and “objective” realities that can be integrated into everyday lives and already existing lifestyles (Jaspal and Cinnirella, 2012).

For example, the same participating family continued explaining how the readily objectified and anchored concept then works in practice, and then could be translated into actual behaviors:

“(…) I think every day I liked to have them live lagom because once we sat down and talked about the concept, once we did that I think that was something that then we can say: ‘the reason that we are packing lunches and putting them into these reusable containers is because this is better for our- the planet.’ And ‘remember, we talked about it.’ (Not-2)

This finding supports the idea that the lagom concept helped project participants to adapt to new behavioral patterns through an increase in both action and procedural-readinessoriginating from their self-concept (Oyserman, 2009). It also lends sustenance via Heider’s (1985 p. 32) seminal work in which he states that “[m]otives and sentiments are psychological entities…Mentalistic concepts (…) [t]hat bring order into behavior.” For example:

“It set us up to be more organized and to think more about stuff. You know, now, when I go food shopping I think about ’oh do I buy that in the plastic, do I buy that in the glass? Do I take these vegetables in the bag or not in the bag? So it impacted on everything! I don’t think ’do I do that because [name retailer] not to or [name retailer] made me think not to, or do I actually do it because it is sensible, isn’t it? The way it should be.” (Re-1)

“It puts it to the forefront of your mind. Especially ’cause I’m still on the Facebook group and you see the post for some of the new people all the time which is really useful. And then it’s just in the back of my mind ’oh, one more thing, one more little change’ so yeah, it is definitely sustainable.” (Br-6)

The project operated on the assumption that in order to allow motivations to arise and behaviors to spill over, a certain level of awareness must be given. Raising awareness was mainly nurtured through the interaction between different participants with diverse focus areas and expertise, and a variety of workshop experiences with experts (see Supplementary Data Sheet 1, Appendix A). As a result, participants consciously changed behaviors, a move originating from an increased level of awareness and intrinsic motivation, rather than emerging from externally regulated factors and changes in the environment allowing for little or no agency (cf. Reckwitz, 2002). For example:

“I think we are much more conscious what we spend our money on so we would much rather do things together as a family or experiences and things like that rather than buying things. So that’s awesome.” (Re-2)

“I think for me it meant being mindful about *how* we are using things to try to minimize wastefulness” (Not-2).

Following an increased awareness, the strengthened motivation also resulted in an improved action readiness to enact more PEB in other settings and thus show more behavioral consistency between domains:

“I don’t think there can be [a limit to a lagom lifestyle]. I think it’s just you have to keep reassessing your contribution and how you can make those small changes, note when you go to the supermarket or packaging you’re buying. All of that, you know, do I need to buy the apples in a plastic bag or can I buy the ones that aren’t? (Nor-2)

#### Limits and Barriers

Whereas the research uncovered an improved understanding of how to enable competences to engage in more sustainable lifestyles, the interviews also highlighted several barriers. One of the key obstacles common amongst the interviewees in relation to more sustainable lifestyles seem to be posed by structural factors, or the lack of them:

“Particularly if there aren’t kind of larger social structures in place to encourage to think that way [sustainably]. Umm, so I think a really good example is like recycling. I don’t think people just actively think about doing it unless it’s brought to their intention and then supported. Just as an active process (…).” (Re-3)

The interviewee then continued explaining how a lack of systems of provision (negatively) impacted their capability to engage in more environmental friendly behavioral patterns:

“(…) [r]ight outside our apartment block there was a huge recycling container. We would just take whatever we could recycle downstairs and put it in the recycling containers (…). And it was like a no-brainer because you are walking out of your building anyway or you are walking up the street (…). So there were so many things about that environment there that just helped us to be more conscious of how we were as consumers…and our impact on the environment whereas here there is just so little of that.” (Re-3)

### Summary

The thematic analysis highlighted the importance of providing an entry point to more sustainable lifestyles such as a behavior change project. It showed that the interaction between a *Lifestyle Change Support System* and a household can change behaviors for an extended time and facilitate positive spillover effects. It also provided participants with important opportunities to raise awareness, rethink traditional ways of living and how to potentially (re-) organize one’s everyday life. Study 2 helped to shed further light on findings from Study 1.

For example, the qualitative analysis showed that interviewees’ initial behavior changes spilled over to other behaviors. At the same time, participants also highlighted factors which continue to cause barriers to engaging in further pro-environmental barriers. Especially *external* factors participants had little control over such as missing infrastructure to commute more sustainably or recycle better seem to cause seemingly insurmountable lock-ins.

Indeed, the main motivation to engage in the Live Lagom project was to receive support to overcome barriers to more sustainable lifestyles. The motivation that resulted from the participation in the project can be linked to the continuous interaction with other participants who allowed each other to explore further PEB. Moreover, the increase in awareness operated as an additional motivator.

Ascribing PEB to their sense of self seems to be a fundamental prerequisite for positive spillover effects and, more generally, in the process toward more sustainable lifestyles. By anchoring a previously unknown concept and attributing (shared) meaning to it, Lagom, became a synonym for sustainable living.

In summary, the findings suggest that new capabilities emerged through the support offered by the Lifestyle Change Support System. This, together with an emerging sense of commitments through an increase in awareness of sustainability related issue, a strengthened sense of belongingness resulting from the interaction with other participants, and a supportive context led to more autonomously motivated PEB enactment that were *not* controlled through external mechanisms such laws. This can be of great importance since previous research has shown that fully integrated, intrinsically motivated behaviors are more stable over time (Hagger et al., 2006). As a result, PEB were explored, enacted and maintained and had the opportunity to spill over.

## General Discussion

The overall aim of the research was to investigate ways that support spillover processes toward crucially needed sustainable lifestyles. Using a mixed-methods approach, the study went beyond purely correlational studies to allow for wider insights, and lend explanatory power to quantitative findings.

We aimed to examine whether participating in a yearlong project ran by a commercial retailer could change PEB and promote positive spillover effects. Moreover, we examined whether behavior changes were associated with (changes) in identity and perceptions of engaging in sustainable lifestyles to be easy and affordable.

The quantitative study 1 showed that behaviors changed. As so often, finding evidence of strong positive spillover effects turned out to be difficult.

The qualitative study 2 interviewed a subsample 9 months later. In this study we found evidence of reported positive spillover effects. Findings suggest that this may be because of the interaction with the *Lifestyle Change Support System* which provided participants with ongoing instrumental and social support, as well as motivational encouragement facilitating capabilities and commitments participants need to adopt changes in behavior.

### Pro-environmental Behavior Change and Spillover Effects

As expected, Study 1 found that respondents in the experimental group were significantly more likely than respondents in the control group to report a change in behavior change. The interventions offered as part of the support from the commercial retailer were thus considered to be successful. However, we were not able to clearly show any evidence of positive or negative spillover. Indeed, the lack of consistency suggests that spillover processes were unlikely.

Another is offered by the qualitative analysis. Study 2 in particular showed that, in order to allow for truly far-reaching behavior changes that do not stop at the foot-in-the-door stage (Thøgersen and Noblet, 2012), a number of supporting factors are needed. Findings suggest that a lack of continuous motivation or capabilities to autonomously enact other PEB is a determinant of positive spillover effects. It is important to note, however, that motivations differed among participants. This can be because the project was not purely framed along the lines of only pro-environmental motivated goals but also intended to show financial incentives. This might have resulted in a lack of causal clarity (Thøgersen and Crompton, 2009) and have even diminished the overall probability of positive spillover effects to occur In addition to that, (non-) existing structural factors such as recycling facilities or missing public transport can lead to inconsistencies of PEB and disallow positive spillover effects.

### Enabler of Behavioral Spillover Effects

#### Sustainable Identity

In line with previous studies (e.g., Whitmarsh and O’Neill, 2010), the quantitative analysis found that environmental identity at time 1 was a significant predictor of behavior change. Yet, the analysis found only a small effect between an increase in pro-environmental identity in the experimental group and spillover effects. One potential explanation for this is that people hold negative stereotypes of environmentalists such as militant, aggressive, unconventional, and eccentric (Bashir et al., 2013) so that participants preferred to be seen as ‘normal’ rather than as sustainable citizens. Moreover, identities are highly relational and context-dependent (Strannegård and Dobers, 2010) allowing people to adapt a more sustainable identity in one context while behaving unsustainable in another.

Here qualitative study 2 found that participating households were often motivated and benefitted from the ongoing interaction with the supporting retailer and the like-minded people. This lead to the adaptation of a pro-environmental mindset operating as a framework for everyday behaviors rather than a prescribed identity.

#### Pro-environmental Capabilities

Contrasting quantitative with qualitative findings, it became obvious that capabilities to engage in further PEB were developed through the interaction between households and *Lifestyle Change Support System*, and households and other participating households. Interestingly, PEB followed the development of competences for sustainable lifestyles rather than through eliminating barriers such as time and money.

The study thus also adds to the understanding of how companies can serve as a force for good by operating as what has been named somewhere else as ‘systems of provisions’ (Spaargaren and Van Vliet, 2000) or, what we call a Lifestyle Change Support System for citizens that goes beyond a purely exchange relationship and that has the potential to fill in an important role in society. Instead a Lifestyle Change Support System needs to provide resources, knowledge and means to help and to act on people’s behalf to secure desired outcomes on the one hand, and to allow people to enact PEB on their own, or, in other words autonomously.

However, and more generally, findings show that humans have very different needs (Amel et al., 2017). For example, whereas some individuals might strive to gain a stronger feeling of belonging to motivate them to engage in further PEB, others might strive to learn more to build more skills and competences, to autonomously enact PEBs.

### Limitations and Directions for Future Research

#### Limitations

One obvious limitation is the small number of interviewees. Whereas this is common in qualitative research studies using interview data, qualitative findings as part of the mixed-methods approach are much more equipped to serve as tool helping to make further sense of the quantitative results.

Moreover, as a real-life experiment the research faces a number of limitations such as making causal inferences between the great number of applied interventions and their specific impact on behaviors. The project involved a lot of different elements, this makes it difficult to assess exactly what the working ingredients are of the intervention. The qualitative data, however, suggest that this broad approach may have been key to its success providing broad support and a sense of belonging.

Drawing on findings from a sample recruited across a number of locations it is difficult to make wider conclusions due to potentially significant regional differences in behaviors based on differing laws, cultures and structural factors (Rentfrow et al., 2015). However, based on the findings there is no reason to assume that responses to the intervention would have been significantly different between demographic areas.

Lastly, both studies also had predominately female participants. According to Scannell and Gifford (2013), women enact more PEB than men so that findings must be interpreted with care when trying to make wider generalizations. Although we did not find significant differences between participating male and female respondents in the quantitative study future research may want to focus on male householders in more detail.

#### Future Directions for Research

Taken together, whereas psychology undoubtedly plays a major role in people’s transitions toward a more sustainable lifestyle (Gifford, 2008), to master today’s challenges posed by climate change and increasing environmental degradation, new mechanisms need to be in place to facilitate more sustainable lifestyles (Gatersleben et al., 2012). Following the Lewinian notion that, in the end, behavior is a function of organism and environment (Stern et al., 1999), and other studies in the field pointing to the importance of contextual and environmental factors (e.g., Lorenzoni et al., 2007; Oyserman and Lewis, 2017), future spillover studies will need to look much closer at contextual and other supporting factors.

Further research is necessary for exploring intervention projects that draw on our findings. In addition, future research can benefit from paying close attention to research in the field of human motivations and using established theories such as self-determination theory (SDT; Ryan and Deci, 2000). Initial studies informed by SDT have shown first promising insights (Webb et al., 2013; Whitmarsh et al., 2017). Lastly, more research is needed in order to examine how an improved customer-company relationship can facilitate autonomy supportive environments that allow for positive spillover effects to occur.

## Conclusion

Lacroix and Gifford (2018) recently asked in a paper: “Can some psychological barriers be eliminated? If a barrier is eliminated, do spillover or, alternatively, rebound effects occur?” Although the participation in the project led to an overall positive shift in perception toward affordability and ease regarding the enactment of PEB, and thus an increase in capabilities, it did not lead to an increase in (positive) spillover effects. Instead, findings from the semi-structured interviews show that especially the interaction and a strong sense of relatedness between the Lifestyle Change Support System and with other households played an important role in facilitating competences and, eventually, to build capabilities allowing for wider lifestyle changes.

Moreover, in the light of recent debates about the potentially necessary degrowth of the consumer economy (e.g., Borowy and Schmelzer, 2017), a project such as this points to the scope for lifestyle change projects to contribute to radical shifts in lifestyle that enable participants to save money and reduce impacts. Moreover, the study indicates the potential of a positive relationship between a company and its customers that goes beyond the usual exchange relationship. At a time of increasing influences from the private sector on citizens it seems a matter of urgency to create more inclusive avenues that are able find ways to co-create sustainable lifestyles. Finally, the research adds to the existing body of spillover effects. In particular, it suggests new insights concerning the ways in which groups can positively influence PEB change. It shows how citizens’ capabilities commitments for sustainable living can be enhanced by a supportive environment enabling identity adaptation. We consider this an exciting area for further research and practical exploration.

## Ethics Statement

Please find below a short version of the ethics application for the Live LAGOM Project which was reviewed and received approval from the University of Surrey Ethics Committee.

### Ethics Application

In September 2015 IKEA launched a major 3-year program of action research as part of its overall strategy for sustainable production and consumption. Its aim is to enable households to live more sustainable lifestyles at home. The Practitioner Doctorate Student (PDS) and his research team made sure that all data collection procedures were in line with the University of Surrey ethics guidelines at all times to allow that the ethics application at hand applies for ethical approval to use data already collected by IKEA in year 1, as well as to all data collection over for the remaining 2 years of the Live LAGOM project until mid-2018.

### Description Live LAGOM Project

The Live LAGOM project is an experimental program that fits into two larger contexts. First, it is part of a major international strategy by IKEA to become a leader in sustainable retail. Second, it reflects a sense that there remains a wide gap between the urgent need for more sustainable living and the response to date from businesses and citizens to the challenges of unsustainable development. The Live LAGOM project aims to generate insight into how barriers to more sustainable living can be overcome. More broadly, the project can be seen as a major opportunity for action research that builds on insights from academic work on sustainable lifestyles, behavior change and values, at the University of Surrey and elsewhere.

The role of CES at the University of Surrey in this project is primarily to analyze the data collected, to write reports and published papers in academic journals, but also to address on methodological issues as appropriate. The project is run by a small team that is part of the IKEA Sustainability Department, with support by the charity Hubbub UK (Hubbub UK is a charity working on a range of pro-environmental and social projects with links to behavior change. For further information please see www.hubbub.org.uk). At the end of the project the PDS and his research team at CES team will provide a report on the evaluation of the overall effectiveness of the project in the context of other behavior change projects.

### Project Methodology

At the beginning of the start of each year, of the 3-years project in October, respectively, it starts with the recruitment of a pool of participating customers.

Participants (c.100 – c.125 per year) are recruited through the IKEA Family data base that formed the experimental group. In the pilot year/exploratory phase participants received a baseline questionnaire in paper form at an in-store workshop and an online follow-up questionnaire. In years 2 and 3 all questionnaires are now online questionnaires, prepared and collected in and through Qualtrics.

At the beginning of January, all participants received a £500 voucher (NB: in year 2, which is the basis for the paper at hand, it was reduced to £300) with which they purchased products from the IKEA sustainability range. This range of products is designed to help participants to live a more sustainable lifestyle, in other words, the products aim to help (i) save energy and water, and/or (ii) improve recycling and upcycling behaviors, and/or (iii) eat healthier, and/or (iv) live more active lifestyles.

Over the course of the project participants will experience a number of additional interventions such as:

(a) monthly newsletters with awareness raising information, among others;
(b) exchange of information on the project Facebook group (private group for participants);
(c) regional events (two to three over the course of the project) that will help participants to build a network and help to stay engaged and receive further inspiration;
(d) support in the form of Q&As or newsletter posts from experts working in different fields of sustainability.

The interviews will take place in the United Kingdom and are conducted by the PDS and, potentially, a member of his research team at the University of Surrey. Depending on the availability of the participants and further conversations with IKEA the research team might conduct interview in Ireland. If this is the case, an updated version of the ethics application at hand will follow in line with Ethics Handbook Section 2.4 on research conducted outside the United Kingdom.

All interviews will be audio recorded and transcribed in line with the University ethics guidelines.

### Analysis

With regard to quantitative data, we use semi-structured questionnaire that will be updated depending on the requirements of the research. All questionnaire responses are marked with a unique identifier (four digit code) before they were collected and safely stored by the PDS according to the University guidelines. All participants’ names were deleted to ensure that they remain anonymous and able to speak freely about their experiences. University of Surrey – Ethicsv.7, November 2015.

## Author Contributions

PE, BG, and IC conceived of the overall approach and structure of the manuscript presented here. PE and BG developed the theory. PE collected the data and wrote the manuscript with support from BG and IC. PE carried out the data collection and conducted the semi-structured interviews and its analysis. BG performed the computations. IC and BG verified the analytical methods and IC supervised the findings of this work. All authors discussed the results and contributed to the final manuscript.

## Conflict of Interest Statement

The studies reported in this publication were funded by the commercial retailer. In line with the agreement between research institution and funding institution, a fully objective examination of the project will be given. As a result, there is thus no conflict of interest given.
