# Supplementary material for: Facilitating Positive Spillover Effects: New Insights From a Mixed-Methods Approach Exploring Factors Enabling People to Live More Sustainable Lifestyles
Source: Front Psychol. 2019 Jan 31;9:2699. doi: 10.3389/fpsyg.2018.02699 (PMC6371024; doi:10.3389/fpsyg.2018.02699)
Supplement: Supplementary file 1 [file Data_Sheet_1.docx]

Appendix A. Intervention overview Live LAGOM project

| Intervention | Type | Definition | Description |
| --- | --- | --- | --- |
| Live LAGOM Brochure | Education  Modelling | Increase knowledge and awareness.  Provide example to aspire to | Information and product brochure provided to explain and showcase products and their potential benefit for the participants. The brochure includes an overview of previous participants and their stories |
| Products | Enablement  Incentivisation  Environmental restructuring | Reduce barriers and create capabilitites  Create expectation of reward.  Changing physical or social context | Rather than providing a purely financial incentive, participants were then allowed to spend up to £300 on a carefully selected product range identified that can help participants to live more sustainable lifestyles at home (e.g. shower-timer, LED light bulbs, energy efficient appliances, among others). |
| Home visit | Enablement  Training | Reduce barriers & create capabilitites.  Imparting skills | A home visit was conducted prior to the project by two IKEA project members that aimed to identify the areas at home where the respective participant could make the greatest advancements towards a more sustainable lifestyle at home |
| Initial meet-up | Enablement  Training | Reduce barriers & create capabilitites.  Imparting skills | An initial meet-up at the retailers’ facilities allowed participants to get to know their local project group, better understand the aim of the project and set themselves goals for their participation. It allowed participants to get to know their contact person at the retailer and build momentum to start the project fully motivated and increase commitment. |
| Workshop 1: Cosy homes | Education  Enablement  Training | Increase knowledge and awareness.  Reduce barriers & create capabilitites  Imparting skills | The first workshop held at the retailers’ respective facilities aimed to develop more skills on how to make one’s home more energy efficient. Moreover, it served as an opportunity to further group together to strengthen both a group identity and establish norms. |
| Workshop 2: Get growing | Education  Enablement  Training | Increase knowledge and awareness.  Reduce barriers & create capabilitites  Imparting skills | The second workshop held at the retailers’ respective facilities aimed to develop skills on how to grow own food at home. Moreover, it served as an opportunity to further group together to strengthen both a group identity and establish norms. |
| Workshop 3: Fermenting | Education  Enablement  Training | Increase knowledge and awareness.  Reduce barriers & create capabilitites  Imparting skills | The third workshop held at the retailers’ respective facilities aimed to develop more skills with regards to avoiding food waste. Moreover, it served as an opportunity to further group together to strengthen both a group identity and establish norms. |
| Food waste challenge | Incentivisation  Modelling | Create expectation of reward.  Provide example to aspire to. | An online food waste challenge intended to allow people to compete with each other to come up with new ideas of how to waste less food. The winner received an incentive provided by the retailer. |
| Online Energy Q&A | Persuasion  Training | Stimulate action through communication  Imparting skills | Participants were contacted to submit their questions regarding energy savings at home. An online live question & answer session with an industry expert was then organised to answer them. |
| Closed Facebook group | Environmental Restructuring | Changing physical or social context | A closed Facebook group was created during the first year. |
| Reflective blog writing | Enablement | Allow participants to reflect on personal progress | Participants were asked to write three blog posts at different points in time (i.e. beginning, mid, end). This was part of the qualitative data collection (NB: Findings are not reported here) and served also as opportunity for the participant to reflect on their progress. |

Appendix B: Changes in reported pro-environmental behaviour by interview participants^1^.

|  |  | Participants | | | | | | | | | | | |
| --- | --- | --- | --- | --- | --- | --- | --- | --- | --- | --- | --- | --- | --- |
|  |  | Re-1 | Re-2 | | Re-3 | | Br-4 | | Br-6 | | Nor-2 | | Not-1 |
|  |  |  | | | | | | | | | | | |
| Home | …switch off lights | (3) +2 | | (5) 0 | | (4) +1 | | (5) 0 | | (4) +1 | | (4) +1 | (5) -1 |
|  | …switch off appliances | (2) 0 | | (2) 0 | | (2) +1 | | (4) 0 | | (3) 0 | | (4) 0 | (2) +2 |
| Home | … maintain, repair and/or "upcycle" things | (4) 0 | | (4) 0 | | (4) 0 | | (4) 0 | | (2) +1 | | (4)+1 | (4) +1 |
| Home | … avoid food waste | (4) +1 | | (5) 0 | | (5) 0 | | (5) 0 | | (2) +2 | | (5) -4 | (4) +1 |
| Non-home: | … use reusable shopping bags? | (4) +1 | | (3) +1 | | (5) 0 | | (5) 0 | | (5) 0 | | (5) 0 | (4) +1 |
| Non-home: | … use energy/ water labelling | (2) +2 | | (1) 0 | | (5) 0 | | (4) +1 | | (2) +1 | | (3) +2 | (4) -1 |
| Non-home: | … choose fairly-traded, eco-labelled | (2) +2 | | (1) +1 | | (4) -1 | | (3) +1 | | (3) 0 | | (2) 0 | (3) 0 |
| Non-home: | … buy second hand or recycled | (4) 0 | | (2) 0 | | (2) +2 | | (4) 0 | | (2) +1 | | (2) 0 | (2) +1 |
| Non-home: | … hire, share and lend products | (2) 0 | | (3) -2 | | (2) +1 | | (3) 0 | | (2) 0 | | (1) +1 | (3) +2 |
| Non-home: | … walk or take the bike instead of the car | (3) +1 | | (3) -1 | | (2)+2 | | (4) 0 | | (2) +1 | | (3) +2 | (5) -1 |
